# A New Remote Monitoring System: Evaluation of the Efficiency and Accuracy of the Smart Emergency Medical System-Health Internet of Things Device

**DOI:** 10.31661/gmj.v13i.3376

**Published:** 2024-08-29

**Authors:** Mohammad Taghi Hedayati Goudarzi, Hadi Zare Marzouni, Fazel Tarkhan, Ali Bijani, Mehdi Babagoli, Amirhossein Shadifar, Javad Abbas Alipour

**Affiliations:** ^1^ Cardiology Department, Rohani Hospital, School of Medicine, Babol University of Medical Sciences, Babol, Iran; ^2^ Qaen Faculty of Medical Sciences, Birjand University of Medical Sciences, Birjand, Iran; ^3^ Biomedical and Microbial Advanced Technologies Research Center, Health Research Institute, Babol University of Medical Sciences, Babol, Iran; ^4^ Department of Epidemiology, Social Determinants of Health Research Center, Health Research Institute, Babol University of Medical Sciences, Babol, Iran; ^5^ IT Engineering Group, Department of Industrial Engineering, K.N. Toosi University of Technology, Tehran, Iran; ^6^ Department of Electrical and Computer Engineering, Faculty of Electrical Engineering, Babol Noshirvani University of Technology, Babol, Iran; ^7^ Clinical Research Development Unit of Rouhani Hospital, Babol University of Medical Sciences, Babol, Iran

**Keywords:** Cardiovascular Disease, Telemedicine, Telehealth, Arrhythmias

## Abstract

Background: The remote medical monitoring system can facilitate monitoring patients with cardiac arrhythmia, and consequently, reduce mortality and complications in individuals requiring emergency interventions. Hence, it is necessary to evaluate new telemedicine devices and compare them with standard devices. Therefore, this study aimed to evaluate and compare the new remote monitoring system, Smart Emergency Medical System-Health Internet of Things (SEMS-HIOT) developed by the Health Technology Development Centre of Babol University of Medical Sciences on patients with different cardiac arrhythmias and compare it with the standard device. Materials and Methods: In this case-control study, 60 patients were divided into the six most common arrhythmia groups (n=10 per each group and equal gender) as atrial fibrillation, ventricular tachycardia, paroxysmal supraventricular tachycardia, premature ventricular contractions, atrial tachycardia, and premature atrial contractions. Also, 20 healthy individuals (including 10 men and 10 women) without any arrhythmia (normal rhythm) were considered as the control group. Three similar SEMS-HIOT devices were used as test devices and a standard cardiac monitoring device as the control device. The clinical parameters, including heart rate, pulse rate, oxygen saturation, body temperature, and cardiac electrical activity via electrocardiogram (ECG) lead-II were recorded. Results: Findings showed that the performance of the SEMS-HIOT test device was similar and in the same range for all indices in each group and there were no significant differences compared to the performance of the control device (P0.05). Also, the ECG records measured with SEMS-HIOT and standard device indicate no significant differences (P0.05). Conclusion: Our study showed that the cardiac indices as well as ECG findings, which were measured with SEMS-HIOT and common standard devices confirmed the accuracy and reliability of the new telematics device for monitoring patients with cardiac diseases.

## Introduction

Cardiovascular diseases (CVDs) are an important health issue that are considered as one of the most important leading causes of death with approximately 20 million cases worldwide annually [1][2][3][4][5]. CVDs include a broad range of disorders and conditions, including coronary heart disease, cardiac arrhythmia, angina pectoris, heart failure (HF), myocardial infarction, hereditary heart disease, congenital heart defects or diseases, and valvular heart disease [6][7].

Current studies have identified various causes for CVDs, including high blood pressure, atherosclerosis, radiation therapy, smoking, poor sleeping habits, unhealthy diet (i.e., high-reached fat, carbohydrates, and cholesterol), obesity, stress, diabetes, low physical activity, and excessive alcohol consumption [7][8]. Although there are various treatments for CVDs, monitoring, follow-up, and regular examination of patients by specialists is an important and necessary principle to control and improve the disease [9][10].

In the past, doctor-patient communication was face-to-face and depended on both being in the same place at the same time, but in line with the rapid development of science and technology, the shape and content of communication and its information have changed [11][12]. Thus, since 1990, following the widespread use of the Internet, new forms of communication between the healthcare team and the patient have been established [13].

The term telemedicine (or telehealth) refers to technology-based virtual programs that can provide various aspects of health information, disease prevention, monitoring, care, etc [14]. Also, remote medical care can be provided through an electronic system based on mobile cell phones and/or other devices [15].

In recent years, several studies have been conducted on the use of remote therapy devices and their effectiveness [16][17]. Evidence indicates that the application of these devices could significantly reduce the need for acute care (such as admission to the emergency department and receiving vital measures), and to register and, or transfer the biometric information of patients (such as heart rate, blood pressure, etc.) [18].

The telemedicine device(s) could have unique features, such as small and portable monitoring, real-time monitoring of the heart's electrical activity in one lead (D2), body temperature and oxygen, movement and position of the person's body in the environment, analysis, and processing of the relationship between vital signs and patient's conditions, and need to emergency services [19][20].

Indeed, in situations where a person needs emergency interventions, the system acts intelligently and informs the emergency medical team and the patient's relatives, consequently, reducing mortality and morbidities [21]. Therefore, a comprehensive evaluation is necessary to examine different telemedicine devices and compare them with each other. However, there are no previous studies regarding remote devices for patients with cardiac diseases in Iranian patients in terms of telemedicine with advanced features. Hence, this study aimed to investigate the efficiency and accuracy of the Smart Emergency Medical System-Health Internet of Things (SEMS-HIOT)– as a new remote therapy device̶ and compare it with the common standard device on individuals with different arrhythmias.

## Materials and Methods

**Figure-1:**
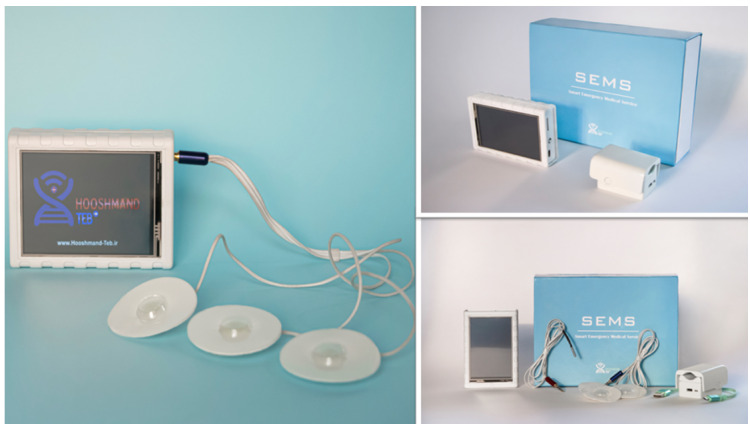
Figure 1. The SEMS-HIOT device

Participants and Study Design

This case-control study was conducted on 80 individuals who attended to the cardiac clinic affiliated with Babol University of Medical Sciences, Babol, Iran during 2023.

Initially, all the participants were evaluated by two cardiologists independently and divided into six most common arrhythmia groups (n=10 per each group and equal gender) as P1 (with atrial fibrillation [AF]), P2 (with ventricular tachycardia [VT]), P3 (with paroxysmal supraventricular tachycardia [PSVT]), P4 (premature ventricular contractions [PVCs]), P5 (atrial tachycardia [AT]), and P6 (premature atrial contractions [PACs]). Also, 20 healthy individuals (including 10 men and 10 women) without any arrhythmia (normal rhythm) were considered as the control group (H) [22].

Data Collections

In this study, a new remote medical care device, named SEMS-HIOT developed by the Health Technology Development Centre of Babol University of Medical Sciences in cooperation with Hooshmand Teb company (Iran) was evaluated. The SEMS-HIOT device (Figure-1) is a portable device that can monitor cardiac features and send them via the internet (through a sim card) and/or wireless to the medical system record center and medical staffs.

To check the repeatability and accuracy of the device, in each group, the clinical information of the patients, including heart rate (HR), pulse rate (PR), oxygen saturation (SPO2), body temperature, and lead-II electrocardiogram (ECG) findings, were collected by four devices including three SEMS-HIOT devices (named D1, D2, and D3) and one standard cardiac monitoring device (Zagros S, Saadat Company, Iran) as the control device (CD). In addition, two cardiologists checked the accuracy and interpretation of the ECG findings.

Ethical Considerations

All the procurers of the current study were reviewed and approved by the Ethics Committee of Babol University of Medical Sciences (ethical code: IR.MUBABOL.REC.1401.180). Also, written informed consent was obtained from all participants for enrollment in the study.

Statistical Analysis

Data were presented as mean and standard deviation (SD), and analyzed by SPSS 21 (IBM, Armonk, NY, USA) using chi-square and one-way ANOVA tests for categorical and numerical variables, respectively. A P-value less than 0.05 was considered as significant statistical difference.

## Results

**Table:** Table1. The Age of Participants In Each Group

		Age				
Groups	Gender			Total		P-value
		Mean	SD	Mean	SD	
AF	Male	54.6	12.49	60.7	11.22	
	Female	66.8	4.74			
AT	Male	50.62	7.7	51.09	9.51	
	Female	46.65	15.7			
PSVT	Male	36.6	6.61	42.88	11.81	
	Female	49.15	12.56			
PVC	Male	38.4	14.67	40.7	12.86	<0.05*
	Female	43	10.38			
PAC	Male	65.8	12.27	60.8	14.22	
	Female	55.8	14.38			
VT	Male	50	10.04	46.8	12.24	
	Female	43.6	13.44			
Control	Male	42.28	9.52	44.54	9.3	
	Female	46.8	8.53			
Total	Male	47.57	13.94	49.01	13.56	0.813
	Female	50.44	13.04			

^*^ Significant comparison between each group with others

In this study, 80 participants with a mean age of 49.01±13.56 years were enrolled. As shown in Table-1, there was no significant difference between the arrhythmia groups compared to the control group in terms of age (P>0.05).

**Figure-2:**
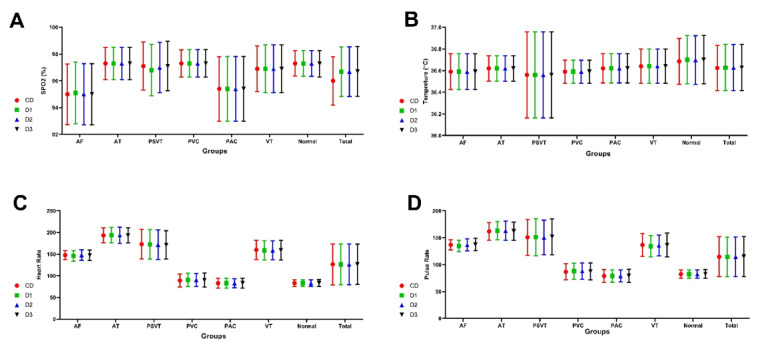
Figure 2. The SPO2 (A), temperature (B), HR (C), and PR (D) were measured with SEMS-HIOT (D1, D2, D3) and CD devices. Findings revealed that all the parameters are similar between SEMS-HIOT devices as well as CD.

Regarding Figure-2A, the mean SPO2 in the total and normal participants was 96±1.8 and 97.3±95, respectively (P>0.05). Also, measured SPO2 with D1, D2, and D3 devices was highly similar. Data analysis revealed that there were no significant statistical differences in SPO2 measured by SEMS-HIOT devices compared to CD (P>0.05, Figure-2A).

Other parameters, including HR, PR, and body temperature that were recorded by D1, D2, and D3 devices were completely similar. In addition, compared to CD device, no significant differences were observed in HR, PR, and temperature that were measured with SEMS-HIOT devices (P>0.05, Figure-2B to D).

The lead-II ECGs recorded with SEMS-HIOT devices showed no differences with those using CD device (P>0.05). Also, the chi-square test indicated no marked differences between D1, D2, and D3 devices in terms of ECG findings among all the studied groups (P>0.05).

## Discussion

The current study revealed that SEMS-HIOT devices could provide similar data, including HR, PR, SOP2, temperature as well as ECG findings compared to standard monitoring devices. Indeed, SEMS-HIOT has acceptable accuracy for use in the proper conditions. Also, our results indicated that in addition to normal patients, SEMS-HIOT was efficiently applied for monitoring patients with any arrhythmia.

Telemedicine uses electronic communication tools and software to provide clinical services to patients without the need for an in-person visit [21]. Telemedicine technology is commonly used for follow-up visits, preventive care support, telehealth in schools, senior center support, chronic disease management, medication management, specialist consultations, and a variety of other clinical services that can be delivered remotely via secure audio and video communications [22][23]. Indeed, telemedicine could applied as an alternative to face-to-face visits which has many advantages for patients and therapists [23]. The benefits of telemedicine for patients include reduced traveling time and distance costs, reduced interference with the responsibility of caring for children or the elderly, reduced possibility of contracting infectious diseases, reduced anxiety, reduced working holidays, and easier access for individuals, living in remote and rural areas [24][25]. Also, telemedicine could provide some benefits for therapists, including increased income, improved practice efficiency, flexible working hours, better follow-up of patients, fewer canceled visits, and remote care after hospitalization [26][27].

The care of patients with CVDs and their constant monitoring are necessary to control the disease and prevent the risks caused by them [28]. Hence, the identification of new methods and the development of appropriate and high-quality tools can be very important and effective in monitoring these patients [29]. Remote cardiac monitoring systems play a crucial role in managing patients with CVDs, particularly those suffering from conditions like cardiac arrhythmia [30]. These systems utilize advanced technology to continuously track and analyze the patient's heart rhythm and parameters, providing real-time data to healthcare providers for timely intervention and treatment [31].

One of the key benefits of remote cardiac monitoring is its ability to detect irregular heartbeats and arrhythmias that may go unnoticed by the patient [32]. So, via continuous monitoring, healthcare providers can identify abnormal heart rhythms early on, allowing for prompt medical intervention to prevent serious complications such as stroke or HF [33].

Patients with cardiac arrhythmias often require long-term monitoring to track the effectiveness of medications, assess the impact of lifestyle changes, or adjust treatment plans as needed [34]. Remote monitoring systems offer a convenient and non-intrusive method to collect data over extended periods, providing a comprehensive view of the patient's heart health without the need for frequent clinic visits [35].

Moreover, remote cardiac monitoring systems enhance the overall quality of care for patients with CVDs by enabling personalized and proactive intervention based on real-time data [36]. Accordingly, healthcare providers can remotely review and analyze the collected information, enabling them to make informed decisions and adjustments to the patient's treatment plan promptly [37]. Also, by promoting proactive management of CVDs, these systems contribute to better patient outcomes, increased patient satisfaction, and more efficient use of healthcare resources [38].

Walter et al. [39] demonstrated that the application of telemedicine can lead to a reduction of 15% in emergency department visits, 17% in bypass costs, 14% in medication costs, 13% in rehabilitation costs, and 59% in catheterization and angioplasty costs. Also, Gallagher et al. [40] and Abraham et al. [41] found that the use of telemedicine can significantly reduce hospitalizations and improve adherence in patients with HF.

However, Boyne et al. [42] showed that early diagnosis of CVDs via telemedicine tools was not significantly reduce rehospitalization rates.

Current study demonstrate that the SEMS-HIOT device exhibits high accuracy in measuring critical health parameters, including HR, PR, SPO2 levels, and temperature, both in patients with cardiac arrhythmias and in individuals without the condition, when compared to the current standard device.

Indeed, the high degree of similarity observed in the recorded data between the SEMS-HIOT device and the standard device underscores the device's capability to provide accurate and consistent measurements in real time, enhancing the quality of care delivered to patients with CVDs, particularly those with cardiac arrhythmias. This finding is particularly promising as it indicates the potential for integrating this new technology into routine clinical practice for improved management and monitoring of cardiac patients.

One key aspect of the new device's performance is its reliability in monitoring important health metrics consistently over time. This continuous monitoring capability can be particularly beneficial for individuals with cardiac arrhythmias, where timely detection and management of irregular heart rhythms are crucial for preventing adverse cardiac events [43].

Moreover, the new remote monitored device's performance extends beyond just accurate data collection, as it also offers features that enhance the overall patient experience and engagement with remote monitoring [44]. On the other words, user-friendly interfaces, remote data accessibility, and real-time alerts for abnormal readings are among the functionalities that can further improve the device's performance and utility in remote patient monitoring scenarios [45]. These features not only foster greater patient compliance with monitoring protocols but also empower patients to take an active role in managing their cardiovascular health.

Another important aspect of the new device's performance is its scalability and interoperability within existing healthcare systems. Seamless integration with electronic health records and other telemedicine platforms can streamline data sharing and communication between patients, clinicians, and care teams, leading to improved care coordination and clinical decision-making [46].

This study has some limitations. The presence of other heart diseases such as HF, as well as other underlying diseases such as diabetes and chronic infections, were not evaluated among all the patients. Hence, further studies with larger sample sizes and more specific CVDs are recommended.

## Conclusion

The findings of the current study show that the SEMS-HIOT device revealed the same results compared to the standard control device in terms of clinical parameters in patients with different cardiac arrhythmia. Therefore, these findings confirm the accuracy and precision of the SEMS-HIOT device.

## Conflict of Interest

The authors declare no conflict of interest.
